# A Systematic Evidence‐Based Review Regarding miRNA Polymorphisms in Recurrent Implantation Failure

**DOI:** 10.1002/rmb2.12670

**Published:** 2025-07-30

**Authors:** Bogdan Doroftei, Ovidiu‐Dumitru Ilie, Ana‐Maria Dabuleanu, Mara Doroftei, Ciprian Ilea, Sergiu Timofeiov, Anca Bivoleanu, Elena Tataranu

**Affiliations:** ^1^ Department of Mother and Child, Faculty of Medicine University of Medicine and Pharmacy “Grigore T. Popa” Iasi Romania; ^2^ Clinical Hospital of Obstetrics and Gynecology “Cuza Voda” Iasi Romania; ^3^ Origyn Fertility Center Iasi Romania; ^4^ Faculty of Medicine University of Medicine and Pharmacy “Grigore T. Popa” Iasi Romania; ^5^ Department of Surgery, Faculty of Medicine University of Medicine and Pharmacy “Grigore T. Popa” Iasi Romania; ^6^ 3rd Surgical Unit “St. Spiridon” County Emergency Clinical Hospital Iasi Romania; ^7^ Faculty of Medicine and Biological Sciences “Stefan Cel Mare” University of Suceava Suceava Romania

**Keywords:** allele, coagulation, genotype, hormonal, in vitro fertilization, microRNA, recurrent implantation failure, renal, single nucleotide polymorphism

## Abstract

**Background:**

This systematic review aimed to evaluate whether specific single nucleotide polymorphisms (SNPs) in miRNAs are associated with recurrent implantation failure (RIF).

**Methods:**

A comprehensive literature search was conducted across PubMed‐MEDLINE, Web of Science, Scopus, and the Excerpta Medica DataBASE.

**Results:**

The Newcastle‐Ottawa Scale (NOS) yielded an intermediate to high quality, with one study rated with 6 stars, and the remaining four with 7 stars. RIF risk‐related genotypes included miR‐196a, miR‐449b, miR‐34a, miR‐146aCG+GG‐miR‐196a2CC, miR‐149TT‐miR‐196a2CC, miR‐196a2CC‐miR‐499AA, miR‐608GC‐miR‐938CC, miR‐27aAG‐miR‐423CC/miR‐604AG/GG and miR‐34aC>A AA‐miR‐130aG>A GG. Protective combinations included miR‐1302‐3, miR‐631II‐miR‐1302‐3CT, and miR‐938CC‐miR‐1302‐3CT. Protective allele combinations G‐T‐T‐A, C‐T, T‐T‐G, T‐T and G‐C‐A‐G, G‐A‐G, A‐G‐G were less frequent in RIF cases, whereas A‐T‐C, T‐C‐C‐T, T‐C‐T, A‐C‐G‐A, A‐A‐G‐G, G‐A‐A‐A, A‐A‐C‐A and G‐G‐A haplotypes were more commonly associated with increased risk. Notably, miR‐608 GC+CC, miR‐1302‐3 CC, miR‐27a AG+GG, miR‐423 CA+AA, miR‐604 AG+GG, miR‐222 GT+TT, and miR‐34a GA+AA were associated with altered coagulation parameters. Additionally, miR‐222 correlated with decreased creatinine levels, the G>T mutation with elevated follicle‐stimulating hormone (FSH), miR‐34aC>A AA genotype with reduced thyroid‐stimulating hormone (TSH) levels, and CA+AA with increased blood urea nitrogen (BUN) levels.

**Conclusions:**

This systematic review highlights that specific miRNA SNPs and haplotype combinations are significantly associated with either increased susceptibility to or protection against RIF.

## 1 Introduction

The field of human‐assisted reproductive technology (ART) has advanced significantly in the management of infertility, demonstrating improved outcomes over time [1]. These advances are reflected in rising global success rates of pregnancies achieved through ART intervention [2]. Nonetheless, concerns persist regarding the risk of implantation failure (IF), which remains a subject of ongoing debate in clinical and research settings [3]. Among ART procedures, in vitro fertilization‐embryo transfer (IVF‐ET) can result in recurrent implantation failure (RIF) [4], a commonly encountered clinical challenge [5, 6], with an estimated prevalence of up to 10% of IVF attempts [7].

Due to the inconsistent application of the term RIF and the lack of a universally accepted definition, this condition is often variably described. A commonly cited definition refers to RIF as the failure to conceive a pregnancy after at least three transfers of high‐quality embryos [8]. Other guidelines adopt broader criteria, defining RIF as two or more consecutive failed transfers, which may include a cumulative total of 4–10 embryos across multiple IVF‐ET cycles or intracytoplasmic sperm injection (ICSI), whether using fresh or frozen embryos or, in some definitions, at least two transferred blastocysts [5, 6, 9].

Given the limited understanding of the pathogenesis of RIF [10, 11] and its complex, multifactorial etiology [12], comprehensive molecular investigations are necessary to uncover its underlying biological mechanisms [13]. In this context, microRNAs (miRNAs) have emerged as key epigenetic regulators of gene expression. They primarily function by mediating post‐transcriptional gene silencing [14, 15] through binding to the 3′‐untranslated regions (3′‐UTRs) of target mRNAs, resulting in either mRNA degradation or translational repression [14, 16].

A growing body of evidence highlights the essential role of miRNAs in maintaining normal cellular functions [17]. Although miRNAs constitute only about 1%–3% of the human genome, they are estimated to regulate the expression of approximately 30% of human genes [18, 19]. Accumulating data have revealed that miRNAs are intricately involved in fundamental biological processes such as cell proliferation, differentiation, and apoptosis [20]. Notably, dysregulated miRNA expression has been linked to impaired fertility in mammals, including disruptions in human implantation [15].

At the molecular level, altered microRNA activity may contribute to an increased susceptibility to RIF, endometriosis (EMS), and impaired endometrial receptivity (ER) among other reproductive disorders [21]. In light of this, the present systematic review aims to evaluate the quality of existing research and to identify microRNA polymorphisms that are most frequently associated with RIF.

## 2 Materials and Methods

### 2.1 Methodology

This systematic review protocol was developed in accordance with the 2020 Preferred Reporting Items for Systematic Reviews and Meta‐Analyses (PRISMA) guidelines [22].

### 2.2 Ethics Statement

This manuscript did not require approval from an Institutional Review Board (IRB) or consent from any third party, as the research data analyzed were extracted from previously published studies in peer‐reviewed journals.

### 2.3 Sources Inquiry

A comprehensive literature search was conducted across four major academic databases: PubMed‐MEDLINE—United States National Library of Medicine (NLM, 1996), Web of Science (WOS) (Clarivate Analytics, 1997), Scopus (Elsevier, 2004) [23, 24, 25], and Excerpta Medica dataBASE (EMBASE) (Elsevier, 1947). These databases were selected due to their complementary coverage and variation in both quantitative and qualitative indexing [26]. The search spanned from January 1st, 2014, to December 1st, 2024, to capture the most current and impactful evidence available in the field. Search strategies were built using both MeSH (Medical Subject Headings) and Emtree terms. The term ‘MicroRNA’ [MicroRNAs] [MeSH] (2003) was used as a major topic [Majr] and can be identified with the MeSH Unique ID: D035683, Tree Number(s): D13.150.650.319, D13.444.735.150.319, D13.444.735.790.552.500 in combination with ‘Polymorphism’ [Polymorphism, Genetic] [MeSH] (2005)—MeSH Unique ID: D011110, Tree Number(s): G05.365.795 and ‘Recurrent Implantation Failure’. As RIF is not recognized as an official MeSH term, the search string incorporated its abbreviation as a free‐text keyword (accessed on 1.12.2024).

### 2.4 String Strategy

Boolean operators such as ‘AND’ or ‘OR’ or ‘NOT’ were employed to construct the initial search strategy (Search #1) as a single query. Additional combinations were used to refine a second series (Search #2). Full details of the search strings and syntax are provided in Supporting Information S1.

### 2.5 Selection of Studies

Potentially eligible references were imported into Mendeley—Reference Management Software (v. 1.19.8) (Elsevier, 2013), which has demonstrated relatively high accuracy in duplicate detection, with a reported sensitivity and specificity of 93%, comparable to that of Ovid/Rayyan (97%) and Covidence (96%) [27]. The ‘Check for Duplicates’ function was applied and supplemented by a manual screening to ensure consistency across records. The titles ± abstracts were independently reviewed by each contributor. Full‐text articles meeting the inclusion criteria were evaluated by O.‐D.I., A.‐M.D. (C)., S.T., and B.D. Any discrepancies or conflicting assessments were resolved unanimously through discussion and consensus. A summarizing table of the retrieved records is provided in Supporting Information S2.

### 2.6 Objective

To address the primary objective in identifying miR polymorphisms associated with either an increased or decreased risk of RIF, we developed a Patient (P), Intervention (I), Comparator (C), and Outcome (O) (PICO) model. This structured model guided the formulation of the research question and eligibility criteria and is presented in Supporting Information S3.

### 2.7 Extraction of Data

Standard methodological data were extracted by O.‐D.I., E.T., and B.D. from the included studies using a structured tabular form in Microsoft Excel 2010 (Microsoft Corporation, Redmond, WA, USA). The spreadsheet was also utilized for sorting and coding raw data. Extracted variables included: first author's name and year of publication, country, study settings, study design, population and sample size, microRNA, method of detection, and identified microRNA polymorphism(s).

### 2.8 Quality Assessment

O.‐D.I., and B.D. independently evaluated the methodological quality of the included studies using the Newcastle‐Ottawa Scale (NOS) [28], appropriate for the appraisal of non‐randomized study designs. The NOS assesses studies across three domains: Selection (score 0–4), Comparability (score 0–2), Exposure (score 0–3). Quality classification was determined based on the total number of asterisks awarded per item: high (7–9), intermediate (4–6), and low quality (0–3).

### 2.9 Criteria of Inclusion and Exclusion

To be deemed eligible for inclusion, studies were required to present original data, be written exclusively in English, and be published in peer‐reviewed journals, regardless of adherence to the IMRaD structure. Additionally, only studies involving human subjects were considered. All other types of publications and analyses were automatically excluded.

## 3 Results

### 3.1 Final Number of Entries and Inclusion

**FIGURE 1:**
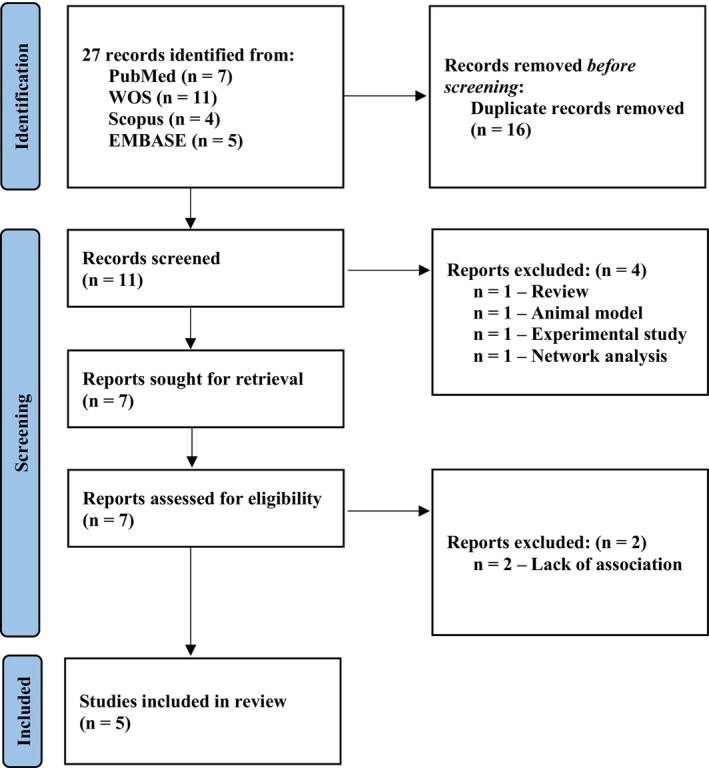
PRISMA flow diagram of the eligible case–control studies.

Out of a total of 27 retrieved records (7 from PubMed‐MEDLINE, 11 from WOS, 4 from Scopus, and 5 from EMBASE), 11 initially met the inclusion criteria for the review after duplicates were removed. During the full‐text screening phase, six studies were excluded due to not meeting our predefined eligibility criteria. The main reasons for exclusion included: use of non‐human (animal) models, studies lacking original data on microRNA polymorphisms associated with RIF, focus on experimental models not directly related to miRNA genetics, and review articles without primary data. Additionally, some studies applied network or bioinformatics analyses without investigating specific single nucleotide polymorphisms (SNPs) or functional impacts on miRNA pathways relevant to RIF. A detailed summary is provided in Figure 1. A complete list of the included manuscripts organized chronologically is provided in Supporting Information S4.

### 3.2 Study Characteristics

The five included studies were published over distinct time periods: one in 2016 [29], two in 2019 [30, 31], one in 2020 [32], and one in 2023 [33]. Most were conducted by researchers based in East Asia, particularly South Korea [30, 31, 32, 33], with collaborations involving Japanese institutions [29] or dual affiliations with universities in the USA [33]. These case–control studies collectively enrolled 1799 women, 1161 controls, and 638 women diagnosed with RIF. Associations between miRNA polymorphisms and RIF were identified using various methods, including a combined approach [29], Polymerase‐Chain Reaction (PCR)‐Restriction Fragment Length Polymorphism (RFLP) [30, 31, 32], or Real‐Time (RT)‐qPCR [33] (Table 1).

**TABLE 1:** Main characteristics of the eligible studies reporting miRNAs polymorphisms in RIF.

Author's first name and year of publication	Country	Study settings	Study design	Population and sample type	microRNA	Method of detection	microRNA polymorphism(s)	References
Cho et al. 2016	South Korea, Japan	*n* = 234* *n* = 120**	Case–control	Blood samples from *n* = 354 women	miR‐146a (rs2910164) miR‐149 (rs2292832) miR‐196a (rs11614913) miR‐499 (rs3746444)	PCR‐RFLP qRT‐PCR	miR‐146aCG+GG/miR‐196a2CC genotype was associated with an increased risk of RIF G‐T‐T‐A (miR‐146a/miR‐149/miR‐196a2/miR‐499) and G‐T‐T (miR‐146a/miR‐149/miR‐196a2) genotype frequencies were less frequent in RIF	[29]
Lee et al. 2019	South Korea	*n* = 212* *n* = 119**	Case–control	Blood samples from *n* = 331 women	miR‐605 (rs2043556) miR‐608 (rs4919510) miR‐631 (rs5745925) miR‐938 (rs12416605) miR‐1302‐3 (rs7589328)	PCR‐RFLP	miR‐938C/1302–3T allele combinations were associated with a decreased risk of RIF T allele of miR‐1302‐3C>T was associated with a decreased risk of RIF miR‐605A/938T/1302–3C allele combination was associated with an increased risk of RIF	[30]
Lee et al. 2019	South Korea	*n* = 228* *n* = 118**	Case–control	Blood samples from *n* = 346 women	miR‐25 (rs1527423) miR‐32 (rs7041716) miR‐125a (rs12976445) miR‐222 (rs34678647)	PCR‐RFLP	miR‐25T/miR‐125aT/miR‐222G was associated with a reduced risk of RIF miR‐25T/miR‐125aT allele combinations were associated with a reduced risk of RIF miR‐25T/miR‐32C/miR‐125aC/miR‐222T allele combinations were associated with an increased risk of RIF	[31]
Kim et al. 2020	South Korea	*n* = 219* *n* = 120**	Case–control	Blood samples from *n* = 339 women	miR‐27a (rs895819) miR‐423 (rs6505162) miR‐449b (rs10061133) miR‐604 (rs2368393)	PCR‐RFLP	miR‐27aA>G, miR‐449bAG+GG, and miR‐604A>G was associated with an increased risk of RIF	[32]
Lee et al. 2023	South Korea	*n* = 268* *n* = 161**	Case–control	Blood samples from *n* = 429 women	miR‐218‐2 (rs11134527) miR‐34a (rs2666433) (rs6577555) miR‐130a (rs731384)	qRT‐PCR	miR‐34aC>A AA genotype was associated with an increased risk of RIF	[33]

* Control group.

** RIF group.

### 3.3 Methodological Heterogeneity and Statistical Considerations

The five studies included in this analysis did not uniformly define RIF, leading to significant heterogeneity. While all studies required the failure to achieve pregnancy after multiple fresh IVF‐ET cycles with embryos that had cleaved into more than 10 cells, notable differences arose regarding the number of failed cycles and the criteria related to both embryo quantity and quality.

Specifically, three studies [29, 30, 31] defined RIF as the inability to conceive after two completed IVF‐ET cycles involving a total of more than 10 cleaved embryos, focusing on a quantity‐based threshold. In contrast, one study [32] applied stricter criteria by requiring failure after two cycles with transfers of only one or two good‐quality embryos, each cleaved into more than 10 cells, emphasizing embryo quality rather than quantity. Another study [33] broadened the definition by requiring failure after more than two IVF‐ET cycles, reflecting a higher severity threshold.

These discrepancies suggest that some studies may have included women with repeated transfers of lower‐quality embryos, while others focused on patients experiencing failure despite transfer of fewer but higher‐quality embryos. This heterogeneity likely influenced patient selection and clinical characteristics across studies, which in turn impacts the strength, comparability, and generalizability of the associations reported with miRNA polymorphisms.

Consequently, these definitional inconsistencies may obscure true genetic effects or limit detection of important subgroup‐specific findings, highlighting the need for cautious interpretation and for standardized RIF definitions in future research.

Additionally, the authors employed a range of statistical methods and software tools to evaluate the association between miRNA polymorphisms and RIF, demonstrating methodological heterogeneity. Most studies calculated sample size and power using QUANTO [29] or G*POWER [30], with a desired power threshold of 80% and a significance level of *p* < 0.05. Genotype and allele frequencies were assessed using Fisher's exact tests or logistic regression, and odds ratios (ORs), adjusted odds ratios (AORs), and 95% confidence intervals (CIs) were calculated.

Three studies used multivariate logistic regression models that adjusted for confounders such as maternal age [30, 31, 32]. However, other key clinical variables such as body mass index (BMI), endometrial thickness, hormonal levels, or number of embryos transferred were either inconsistently reported or not included in the models, limiting the capacity to control for all relevant confounding factors. Hardy–Weinberg equilibrium (HWE) testing was uniformly performed to validate genotype distributions.

To address multiple comparisons, some studies applied Bonferroni correction [29] or the false discovery rate (FDR) approach [30], strengthening the robustness of the reported associations. Combined genotype and haplotype risk scores were typically derived from genotype interaction models, with three studies employing multifactor dimensionality reduction (MDR) [29, 30, 32] to identify synergistic gene–gene effects. However, most studies did not specify whether the cumulative risk was estimated using additive, multiplicative, or interaction‐based models. Only one study explicitly calculated inferred genotype frequencies from MDR results to estimate the combined effect of miRNA variants [29], while the remaining studies reported ORs for genotype combinations without detailing whether a composite or weighted risk score was used. Consequently, the interpretation of cumulative genetic risk remains uncertain due to the lack of standardized or transparent scoring methodology.

Given the limited number of eligible studies (*n* = 5), the potential for publication bias cannot be excluded. Although formal assessments such as Egger's regression or funnel plot asymmetry analysis were not feasible, the possibility of selective reporting should be considered when interpreting these findings. To enhance methodological transparency and improve future meta‐analytic work, future research should adopt standardized statistical modeling approaches, adjust for key clinical confounders, and clearly report the derivation and interpretation of cumulative genetic risk scores.

### 3.4 Quality of Case–Control Studies Assessment

**FIGURE 2:**
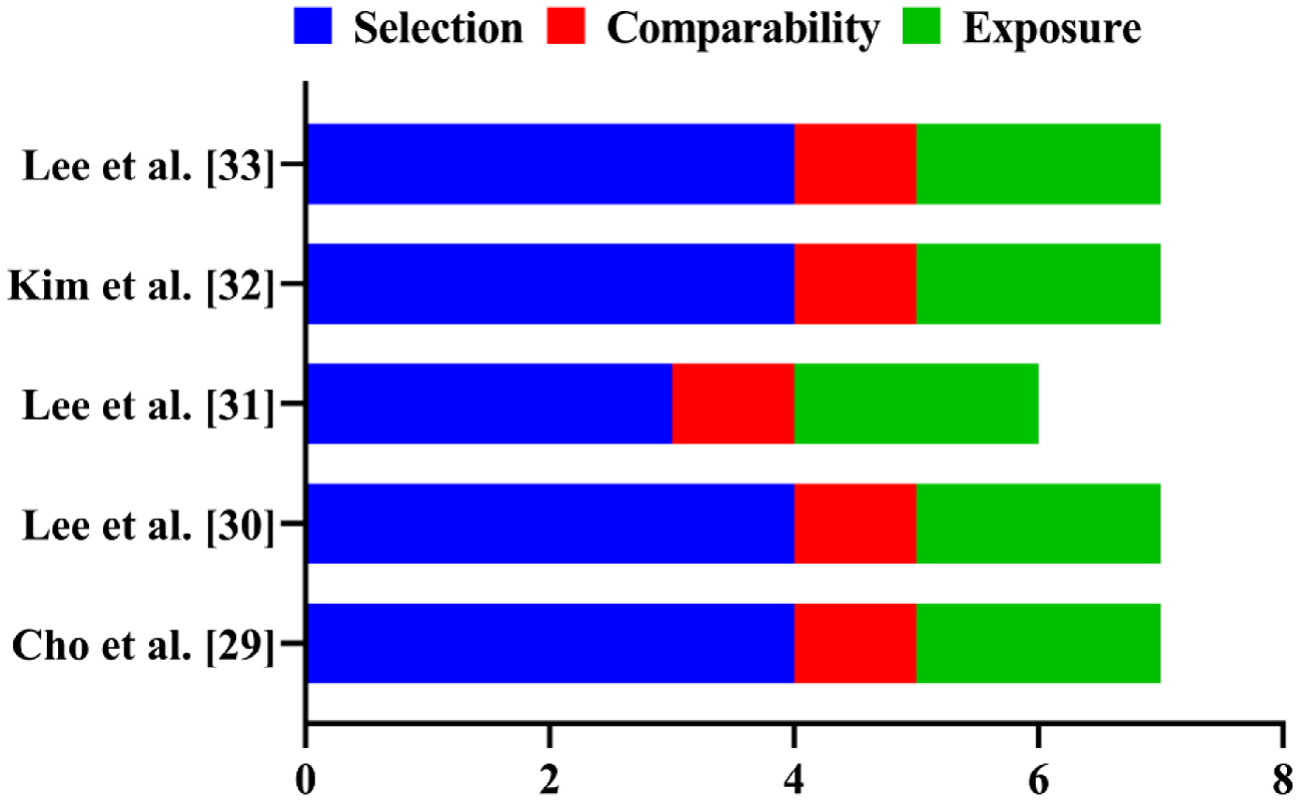
Graphical representation of the overall score following NOS.

As shown in Table 2 and Figure 2, the overall quality of the eligible case–control studies ranged from intermediate to high across all three assessment items, with the exception of Lee et al. [31], which did not adequately characterize the control group under the Selection criterion. In contrast, the remaining four studies provided detailed descriptions of participant enrollment. Non‐RIF control participants were required to have a regular menstrual cycle, a history of at least one naturally conceived pregnancy, no history of pregnancy loss or preeclampsia, and a normal karyotype [46XX] [29, 30, 32, 33]. Regarding the Outcome criterion, a score for the ‘Non‐Response Rate’ could not be assigned; however, the total quality scores were 6 [31] and 7 for the other four studies [29, 30, 32, 33].

**TABLE 2:** Quality assessment of eligible case–control studies based on NOS.

Study	Case definition	Case representativeness	Controls selection	Controls definition	Comparability	Exposure ascertainment	Method ascertainment	Non‐response rate	Total
Cho et al. [29]	*	*	*	*	*	*	*	—	7
Lee et al. [30]	*	*	*	*	*	*	*	—	7
Lee et al. [31]	*	*	*	—	*	*	*	—	6
Kim et al. [32]	*	*	*	*	*	*	*	—	7
Lee et al. [33]	*	*	*	*	*	*	*	—	7

*Note:* “*” represents 1 point within the NOS scale for each item, whereas “—” represents 0 points.

### 3.5 Differential Profile Between Participants

#### 3.5.1 Genotype Frequencies in RIF

IF is widely understood to result from multifactorial biological disruptions, and miRNA polymorphisms have emerged as plausible contributors to this complexity. Variations in miRNA sequences can alter gene regulatory networks, particularly those involved in ER, immune modulation, and hormone signaling. Thus, assessing the frequency of specific miRNA genotypes in women with RIF may help identify genetic predispositions. This section reviews the associations between distinct miRNA genotypes and RIF occurrence, exploring their potential as risk or protective factors depending on IF frequency and individual genetic background. Among the reviewed studies, Lee et al. [31] reported no significant differences in genotype frequencies between RIF and healthy individuals. In contrast, other investigations have indicated either a protective effect [30] or increased susceptibility [29, 32, 33]. For instance, Cho et al. [29] were among the first to observe an association between the miR‐196a2C>T polymorphism and RIF risk, although this was not statistically significant (*p* > 0.05) relative to wild‐type (WT) homozygotes. The AOR was 1.488, with a 95% CI ranging from 0.749 to 2.955, with no major differences in the number of IFs. When considering the combined CT+CC genotype, the AOR was 1.230, with a 95% CI of 0.720–2.099. Kim et al. [32] later demonstrated a significant association between the miR‐449bA>G polymorphism and increased RIF, particularly for the AA genotype compared to AG+GG (AOR = 1.584, 95% CI = 1.008–2.490, *p* = 0.046). The risk increased in women who had experienced at least four IFs. The AG+GG genotype was associated with a higher RIF risk compared to AA (AOR = 1.932, 95% CI = 1.122–3.327, *p* = 0.018). Subsequently, Lee et al. [33] identified the miR‐34aC>A AA genotype as significantly associated with more than two IFs (AOR = 2.264, 95% CI = 1.007–5.092, *p* = 0.048). Consistent findings indicated that patients with this genotype, particularly those with multiple IFs, are at increased risk. Specifically, for women with more than three IFs, the AA genotype had an AOR of 2.322, 95% CI of 1.052–5.125, and *p* = 0.034, while the recessive comparison (CC+CA vs. AA) yielded an AOR = 2.406, 95% CI = 1.068–5.419, *p* = 0.037. With four or more IFs, the AOR increased to 2.783 and 95% CI of 1.185–6.536, *p* = 0.019, and for CC+CA versus AA, the AOR was 2.457, 95% CI = 1.077–5.606, *p* = 0.033, further reinforcing a genetic influence in RIF [33].

Conversely, Lee et al. [30] found that the miR‐1302‐3 CT genotype (AOR = 0.234; 95% CI = 0.089–0.618, *p* = 0.003) was associated with a decreased RIF risk, and this effect persisted with the CT+TT combination; AOR = 0.227; 95% CI = 0.086–0.598, *p* = 0.003. In women with more than three IFs, the CT genotype had an AOR of 0.261 with a 95% CI of 0.099–0.690 and *p* = 0.007, and the CT+TT genotype showed an AOR of 0.253 with a 95% CI of 0.096–0.668, and *p* = 0.006. For four IFs, the CT genotype had an AOR of 0.301 and 95% CI between 0.103 and 0.882, and *p* = 0.029, and the CT+TT combination showed an AOR of 0.292, 95% CI of 0.100–0.853, and *p* = 0.024.

#### 3.5.2 Allele Combinations in RIF

Beyond the effects of individual genotypes, combinations of alleles from different miRNA loci may interact epistatically, impacting implantation‐related processes such as cytokine balance, cellular adhesion, and angiogenesis. Given that gene regulation by miRNAs is cumulative, evaluating haplotypes and combinations of alleles provides a more comprehensive understanding of genetic susceptibility. This section explores the allele combinations that have been reported to influence the risk of RIF, either increasing susceptibility or providing protection. It emphasizes statistically significant haplotypes found in patient cohorts. A comprehensive analysis of the haplotype data across multiple studies has identified several microRNA polymorphisms combinations that appear to modulate susceptibility to RIF. Specifically, certain haplotypes were consistently associated with a reduced risk of RIF. These included G‐T‐T‐A [29], C‐T [30], T‐T‐G, T‐T [31], and G‐C‐A‐G, G‐A‐G, A‐G‐G [32]. Statistically, these associations were supported by Fisher's exact test, followed by Bonferroni or FDR corrections for multiple comparisons. For example, the G‐T‐T‐A haplotype showed a protective effect with an AOR = 0.443, 95% CI = 0.261–0.753, *p* = 0.002; Bonferroni‐adjusted *p* = 0.035 [29], while the C‐T combination demonstrated a similarly reduced risk; OR = 0.259, 95% CI = 0.100–0.674, FDR‐*p*‐value = 0.003 [30]. Protective effects were also observed for T‐T‐G: AOR = 0.528, 95% CI = 0.282–0.990, *p* = 0.044, and T‐T: AOR = 0.510, 95% CI = 0.285–0.913, *p* = 0.022 [31], as well as for G‐C‐A‐G: OR = 0.248, 95% CI = 0.115–0.537, *p* = 0.0001, G‐A‐G: OR = 0.337, 95% CI = 0.174–0.652, *p* = 0.001, FDR = 0.007, and A‐G‐G: OR = 0.094, 95% CI = 0.005–1.636, *p* = 0.030, FDR = 0.210 [32].

In contrast, several haplotypes were found to be significantly associated with an increased risk of RIF. Among the most notable was A‐T‐C (OR = 31.670, 95% CI = 1.802–556.500, *p* = 0.0003) [30]. Other risk‐enhancing combinations included T‐C‐C‐T (AOR = 1.496, 95% CI = 1.000–2.237, *p* = 0.049) and T‐C‐T (AOR = 1.585, 95% CI = 1.071–2.345, *p* = 0.021) [31], as well as A‐C‐G‐A (OR = 2.352, 95% CI = 1.260–4.390, *p* = 0.007), A‐A‐G‐G (OR = 8.818, 95% CI = 1.004–77.460, *p* = 0.030), and G‐A‐A‐A (OR = 5.291, 95% CI = 1.627–17.210, *p* = 0.006) [32]. Additional risk‐associated haplotypes identified included A‐A‐C‐A (OR = 3.687, 95% CI = 1.078–12.61, *p* = 0.034) and G‐G‐A (OR = 2.111, 95% CI = 1.044–4.269, *p* = 0.034) [33].

#### 3.5.3 Combined Genotypes in RIF

Individual miRNAs frequently target overlapping sets of mRNAs, implying combined effects of multiple miRNA polymorphisms that may result in more significant physiological outcomes. These synergistic or antagonistic genotype patterns could influence critical reproductive processes such as trophoblast invasion, endometrial remodeling, and immune tolerance. This section explores how combined miRNA genotypes affect IF, with the aim of identifying profiles that either increase or reduce the risk based on the co‐occurrence of polymorphisms. Cho et al. [29] reported that individuals carrying the combined genotypes miR‐146aCG+GG and miR‐196a2CC have a significantly higher susceptibility to RIF (AOR = 2.022, 95% CI = 1.044–3.916, *p* = 0.037). Other combinations involving miR‐196a2CC, specifically with miR‐149TT (AOR = 1.580, 95% CI = 0.811–3.076) and with miR‐499AA (AOR = 1.449, 95% CI = 0.801–2.621) were also associated with elevated risk, although these did not reach statistical significance. Complementary findings revealed that the miR‐608GC with miR‐938CC genotypes, when combined, were significantly associated with increased RIF risk (OR = 1.811, 95% CI = 1.024–3.202, *p* = 0.041). In contrast, other genotype combinations appeared to confer a protective effect. Notably, individuals carrying both miR‐631II and miR‐1302‐3CT (OR = 0.244, 95% CI = 0.092–0.650, *p* = 0.005) and miR‐938CC with miR‐1302‐3CT (OR = 0.270, 95% CI = 0.101–0.718, *p* = 0.009) had a markedly lower prevalence of RIF [30]. Further evidence supports the role of miRNA combinations in modulating implantation outcomes. The presence of miR‐27aAG with miR‐423CC or miR‐604AG/GG genotype was significantly associated with RIF; (AG/CC: AOR = 0.417; 95% CI = 0.223–0.780; *p* = 0.006, respectively, AG/AG: AOR = 0.329; 95% CI = 0.153–0.706; *p* = 0.004 and AG/GG: AOR = 0.193, 95% CI = 0.054–0.693, *p* = 0.012) [32]. Lastly, the combination of miR‐34aC>A AA and miR‐130aG>A GG genotype as a potential factor was significantly associated with RIF (AOR = 2.881, 95% CI = 1.132–7.332, *p* = 0.026) [33].

#### 3.5.4 Coagulation Factors

In the context of implantation biology, proper regulation of coagulation is essential for establishing and maintaining ER and vascular integrity. Aberrations in coagulation parameters may disrupt this delicate balance and contribute to IF. Several studies have explored how miRNA polymorphisms may influence coagulation profiles in women with RIF, shedding light on a potential molecular link between genetic variability and thrombotic risk in early pregnancy. The outcomes of hematological evaluations aimed at determining the potential risk of blood clots indicated that prothrombin time (PT), activated partial thromboplastin time (aPTT), and platelet count (PLT) exhibited synergistic influences on particular microRNA polymorphisms associated with RIF [30, 31, 32, 33]. The number of PLT in both groups was similar, indicated by the non‐significant difference (*p* > 0.05) [30, 31, 32, 33]. Nonetheless, additional analyses confirm significant differences in PT (*p* < 0.05) per Lee et al. [31, 33], contrasting with earlier studies (*p* > 0.05) [30, 32]. According to the data, similar findings were noted for aPTT in three [30, 31, 32] out of five eligible articles [33], except for the one that concentrated solely on the RIF group [29]. Considering harmful effects, the aPTT showed a significant change (*p* < 0.05) [30, 31, 32] compared to the findings of Lee et al. [33] as indicated by *p* = 0.224. In addition to the aforementioned, specific genotypes showed a strong correlation with RIF across all three coagulation factors. Among the key genotype variants were miR‐608 GC+CC, miR‐1302‐3 CC [30], miR‐27a AG+GG, miR‐423 CA+AA, miR‐604 AG+GG [32], miR‐222 GT+TT [31], and miR‐34a GA+AA [33].

#### 3.5.5 Hormonal Factors

Given that hormone signaling is fundamental to endometrial preparation and embryo implantation, any miRNA‐mediated disruption in hormone levels could plausibly interfere with reproductive outcomes. Recent evidence suggests that miRNA polymorphisms may exert regulatory control over key reproductive hormones, further linking genetic variability to implantation physiology. The analysis of hormones associated with reproduction in RIF individuals revealed significant changes in estradiol (E2) and luteinizing hormone (LH) levels (*p* < 0.05). In comparison to E2 and LH, follicle‐stimulating hormone (FSH) levels were identified to be within the normal limits (*p* > 0.05) [30, 31, 32, 33]. It has been noted that FSH levels could increase depending on the presence of the miR‐222 G>T mutation [31], while thyroid‐stimulating hormone (TSH) is generally found to be reduced in individuals possessing the miR‐34aC>A AA genotype (*p* = 0.003).

#### 3.5.6 Renal Factors

Although renal function may seem tangential to implantation, emerging evidence suggests that systemic metabolic stress, reflected in altered renal biomarkers, can influence uterine receptivity. In this regard, miRNA polymorphisms may play a role in shaping the renal microenvironment and, by extension, the broader physiological milieu necessary for successful implantation. Regarding other predisposing factors that may influence pregnancy outcomes, blood urea nitrogen (BUN) and creatinine levels were higher in RIF patients compared to controls (*p* < 0.0001) [31, 33]. In addition to this distinguishing characteristic, the authors demonstrated that miR‐222 polymorphisms result in a reduction of creatinine levels (*p* < 0.05) [31], whereas the miR‐34aC>A CA+AA genotype was associated with an increased level of BUN (*p* = 0.015) [33].

## 4 Discussion

In this systematic review, we aimed to summarize available evidence from case–control studies regarding the frequencies and combinations of miRNA genotypes and alleles significantly contributing to RIF. Among the five eligible studies, four received a high‐quality score of 7 [29, 30, 32, 33], while one study was rated as intermediate with a score of 6 [31] due to insufficient characterization of control participants' medical histories.

To improve interpretability, Table 3 presents a consolidated summary of the most significant miRNA polymorphisms and haplotype combinations associated with RIF, highlighting the direction of risk, effect sizes (ORs/AORs), and corresponding biological mechanisms reported in the literature. To further elucidate the potential functional impact of these miRNA variants, we compiled a list of both experimentally validated and bioinformatically predicted target genes for the key miRNAs identified in this study. These target genes are implicated in critical biological pathways relevant to RIF, including ER, immune regulation, and hormonal signaling, as summarized in Table 4.

**TABLE 3:** Summary of miRNA polymorphisms and associated risk of RIF with proposed mechanisms.

miRNA genotype/combination	Risk direction	AOR/OR (95% CI)	Proposed mechanism	References
miR‐146a CG+GG+miR‐196a2 CC	↑	2.022 (1.044–3.916)	Inflammatory signaling, ER	[29]
miR‐196a2 CC+miR‐149 TT	↑	1.580 (0.811–3.076)	ER	[29]
miR‐196a2 CC+miR‐499 AA	↑	1.449 (0.801–2.621)	Unknown	[29]
G‐T‐T‐A haplotype	↓	0.443 (0.261–0.753)	Immunomodulatory via NF‐κB	[29]
miR‐1302‐3 CT/CT+TT	↓	0.234 (0.089–0.618)/0.227 (0.086–0.598)	Hormonal regulation, coagulation modulation	[30]
miR‐608 GC+miR‐938 CC	↑	1.811 (1.024–3.202)	Coagulation regulation	[30]
miR‐631 II+miR‐1302‐3 CT	↓	0.244 (0.092–0.650)	Immunological protection	[30]
miR‐938 CC+miR‐1302‐3 CT	↓	0.270 (0.101–0.718)	Protective interaction	[30]
A‐T‐C haplotype	↑	31.67 (1.802–556.5)	Strong genetic susceptibility	[30]
C‐T haplotype	↓	0.259 (0.100–0.674)	Immunological modulation	[30]
T‐T haplotype	↓	0.510 (0.285–0.913)	Genetic protective factor	[31]
T‐C‐T combination	↑	1.585 (1.071–2.345)	Endometrial dysfunction	[31]
T‐C‐C‐T haplotype	↑	1.496 (1.000–2.237)	Unknown	[31]
miR‐222 GT+TT	↑	Not provided	FSH dysregulation	[31]
miR‐449b AA	↑	1.584 (1.008–2.490)	≥ 4 IFs	[32]
miR‐27a AG+miR‐423 CC	↓	0.417 (0.223–0.780)	Hormonal modulation	[32]
miR‐27a AG+miR‐604 AG	↓	0.329 (0.153–0.706)	Hormonal modulation	[32]
miR‐27a AG+miR‐604 GG	↓	0.193 (0.054–0.693)	Hormonal modulation	[32]
G‐C‐A‐G haplotype	↓	0.248 (0.115–0.537)	Genetic protective factor	[32]
G‐A‐G haplotype	↓	0.337 (0.174–0.652)	Unknown	[32]
A‐G‐G haplotype	↓	0.094 (0.005–1.636)	Unknown	[32]
A‐C‐G‐A haplotype	↑	2.352 (1.260–4.390)	Unknown	[32]
A‐A‐G‐G haplotype	↑	8.818 (1.004–77.460)	Unknown	[32]
G‐A‐A‐A haplotype	↑	5.291 (1.627–17.210)	Unknown	[32]
miR‐34a C>A AA	↑	2.322 (1.052–5.125)	Hormonal and renal dysfunction	[33]
miR‐34a C>A AA+miR‐130a G>A GG	↑	2.881 (1.132–7.332)	Hormonal/genetic synergy	[33]
A‐A‐C‐A haplotype	↑	3.687 (1.078–12.61)	Unknown	[33]
G‐G‐A haplotype	↑	2.111 (1.044–4.269)	Unknown	[33]

Abbreviations: ↑, increased risk; ↓, decreased risk; AOR, adjusted odds ratio; CI, confidence interval; IFs, implantation failures; OR, odds ratio.

**TABLE 4:** Key microRNAs and their validated or predicted target genes associated with RIF.

miRNA	Target gene(s)	Validation status	Bioinformatic source(s)	Biological role	Relevance to implantation	Reference(s)
miR‐146a	IRAK1, TRAF6	Validated	miRTarBase, Literature	Inflammatory signaling via NF‐κB	Regulation of endometrial immune balance	[29]
miR‐149	GIT1, FOXM1	Predicted	TargetScan, miRDB	Cell proliferation, migration	Trophoblast invasion, ER	[29]
miR‐196a2	HOXB8, ANXA1, ERG	Predicted	TargetScan, DIANA‐TarBase	Cell differentiation, immune signaling	Endometrial patterning, vascular remodeling	[29]
miR‐608	TP53, IL1A	Predicted	Not consistently predicted in TargetScan/miRTarBase	Inflammation, apoptosis	Immune surveillance, endometrial immune tolerance	[30]
miR‐938	IL6, CXCL10	Predicted	TargetScan, miRDB	Immune response	Modulation of endometrial immune tolerance	[30]
miR‐1302‐3p	ZC3H12C	Predicted	TargetScan	Immune regulation	Cytokine homeostasis and inflammation	[30]
miR‐222	CDKN1B, ERα (ESR1)	Validated	miRTarBase, Literature	Cell cycle, E2 signaling	Affects E2 signaling and endometrial cell receptivity	[31]
miR‐27a	PPARG, VEGFA	Predicted	TargetScan, DIANA‐TarBase	Angiogenesis, hormonal regulation	Vascularization, E2‐mediated endometrial development	[32]
miR‐449b	CDK6, NOTCH1	Predicted	TargetScan, miRDB	Cell cycle regulation, decidualization	Stromal cell differentiation, epithelial receptivity	[32]
miR‐423	PA2G4	Predicted	TargetScan, miRDB	Cell proliferation, differentiation	Endometrial cell growth and receptivity	[32]
miR‐604	FGF2, MMP9	Predicted	TargetScan, DIANA‐TarBase	Extracellular matrix remodeling, cell proliferation	Trophoblast invasion, ER	[32]
miR‐34a	BCL2, SIRT1	Validated	miRTarBase, Literature	Apoptosis, hormonal sensitivity	Control of stromal cell survival, progesterone response	[33]

miR‐146a has been experimentally validated to target key inflammatory signaling molecules IRAK1 and TRAF6, modulating the NF‐κB pathway to maintain a balanced immune environment within the endometrium. This immunoregulatory function is crucial, as an aberrant inflammatory response may lead to rejection of the embryo and IF. Taganov et al. [34] first identified miR‐146a as a negative feedback regulator of NF‐κB signaling via suppression of IRAK1 and TRAF6 in innate immune responses. Further studies demonstrated that miR‐146a attenuates NF‐κB‐driven cytokine production in immune cells [35] and may participate in implantation‐related immune tolerance, as evidenced by altered expression in ART and pregnancy complications [36].

Similarly, miR‐222 targets cell cycle regulator CDKN1B and E2 receptor ESR1, thereby controlling cell proliferation and hormonal signaling that orchestrate endometrial preparation and receptivity. Dysregulation of these pathways could impair E2‐mediated proliferation of endometrial epithelial cells during the implantation window. The pivotal role of miR‐34a in modulating apoptosis via BCL2 and hormonal sensitivity through SIRT1 further underscores the importance of tightly regulated stromal cell survival and progesterone responsiveness in decidualization and embryo acceptance. In support, Mahfouz et al. [37] showed that miR‐34a regulates SIRT1 and FoxO1 expression in endometrial tissue, contributing to reproductive pathology such as EMS.

Beyond these experimentally confirmed interactions, bioinformatic predictions highlight a suite of additional miRNAs potentially involved in implantation biology. For example, miR‐149 is predicted to regulate GIT1 and FOXM1, genes associated with cell proliferation and migration, both of which are critical for trophoblast invasion and endometrial remodeling. Chen et al. [38] demonstrated miR‐149 targeting of GIT1 in breast cancer models, offering mechanistic insights into its role in regulating cell motility and integrin signaling. Likewise, miR‐196a2 potentially targets HOXB8, ANXA1, and ERG, genes implicated in cellular differentiation and immune modulation, suggesting involvement in endometrial patterning and vascular remodeling essential for placental development.

Further predicted interactions highlight the roles of angiogenesis and structural remodeling in implantation. miR‐27a is thought to target PPARG and VEGFA, implicating it in endometrial vascularization necessary for embryo support. Other miRNAs such as miR‐449b, miR‐423, and miR‐604 may influence stromal cell decidualization, epithelial receptivity, and extracellular matrix remodeling via predicted targets like CDK6, NOTCH1, PA2G4, FGF2, and MMP9. These processes are integral to successful implantation. Although miR‐608 and miR‐938 are predicted to target immune‐regulatory genes such as TP53, IL1A, IL6, and CXCL10, their associations remain inconsistent across prediction platforms and lack experimental validation. Nonetheless, their involvement in immune homeostasis warrants further investigation in the context of maternal‐fetal immune tolerance.

miR‐1302‐3p represents a less‐characterized candidate in implantation biology. Although it has been detected in various tissues, including the placenta, its specific function in ER remains largely unknown. Current bioinformatic analyses, such as those from miRDB, have predicted potential target genes for miR‐1302‐3p, but these have not been validated in reproductive tissues [39]. Its limited characterization suggests that future studies are needed to clarify any potential roles in implantation.

Current research identified several miRNAs associated with RIF risk, including miR‐196a [29], miR‐449b [32], and miR‐34a [33], contrasting with miR‐1302‐3 [30], which exhibited differing genotype frequencies. Genotype combinations linked to increased RIF risk include miR‐146aCG+GG‐miR‐196a2CC, miR‐149TT‐miR‐196a2CC, miR‐196a2CC‐miR‐499AA [29], miR‐608GC‐miR‐938CC [30], miR‐27aAG‐miR‐423CC/miR‐604AG/GG [32] and miR‐34aC>A AA‐miR‐130aG>A GG [33]. Conversely, combinations such as miR‐631II‐miR‐1302‐3CT and miR‐938CC‐miR‐1302‐3CT were associated with a reduced risk [30]. Similarly, certain allele combinations were less frequently reported in RIF patients, including G‐T‐T‐A [29], C‐T [30], T‐T‐G, T‐T [31], and G‐C‐A‐G, G‐A‐G, A‐G‐G [32], while other haplotypes such as A‐T‐C [30], T‐C‐C‐T, T‐C‐T [31], A‐C‐G‐A, A‐A‐G‐G, G‐A‐A‐A [32], A‐A‐C‐A, and G‐G‐A [33] showed increased risk.

ER is a complex, pivotal process for the successful embryo implantation [40, 41] regulated by differentially expressed miRNAs [42], especially in RIF [40]. These miRNAs play essential roles in the early stages of pregnancy, including decidualization and placental development [43]. Abnormal miRNA expression has been linked to blastocyst IF [44] and other reproductive disorders [45, 46] by affecting cellular processes such as cell adhesion and junctions [47, 48]. Genes related to RIF are enriched in Wnt signaling [49] and cyclin‐D2‐mediated cell cycle pathways [48], with dysregulation potentially impairing blastocyst implantation [41] and folliculogenesis [50]. MiRNA dysfunction is also implicated in premature ovarian failure (POF) [51], recurrent spontaneous abortion (RSA) [52], and recurrent pregnancy loss (RPL) [53].

Emerging studies reinforce the clinical importance of miRNA polymorphisms and expression profiles in the pathophysiology of RIF. For instance, Park et al. [54] demonstrated that allele combinations such as miR‐25T/miR‐125aT/miR‐222G reduced RIF risk, whereas miR‐25T/miR‐32 C/miR‐125a C/miR‐222T were linked to an increased risk. Notably, the miR‐222 GT+TT genotypes in conjunction with prolonged PT (≥ 12 s) significantly elevated the risk of RIF, highlighting a gene–environment interaction where miRNA variants may influence coagulation pathways critical for implantation.

Functional analyses further elucidate molecular mechanisms. von Grothusen et al. [55] reported downregulation of hsa‐miR‐486‐5p and hsa‐miR‐92b‐3p in uterine fluid of RIF patients, implicating disrupted signaling pathways vital for endometrial preparation and embryo implantation. Similarly, Zhang et al. [56] found decreased miR‐30d‐5p in RIF endometrial samples, with upregulation of its target suppressor of cytokine signaling 1 (SOCS1) and reduced levels of implantation markers such as leukemia inhibitory factor (LIF) and phosphorylated STAT3. These molecular changes may impair pinopode formation and decidualization, ultimately hindering successful embryo attachment.

These findings extend the clinical relevance of miRNA profiling by linking specific polymorphisms and expression changes to coagulation, hormonal, and immune pathways involved in implantation, underscoring the potential for miRNA‐based diagnostics and personalized therapies.

From a clinical perspective, these polymorphisms offer implications beyond diagnostics and may guide personalized therapeutic strategies. For example, variations in miR‐222 [31] and miR‐34a [33] influence FSH and TSH levels, suggesting a potential role for tailored hormonal supplementation. Patients with miR‐222 G>T variants exhibited elevated FSH [31], and those with the miR‐34a AA genotype showed decreased TSH [33], both of which could contribute to suboptimal ER and support the rationale for endocrine profiling and individualized hormonal therapy during IVF cycles.

Additionally, several polymorphisms correlate with alterations in coagulation markers (PT, aPTT), which may warrant personalized thromboprophylaxis. Variants such as miR‐608GC+CC, miR‐1302‐3CC [30], miR‐27a AG+GG [32], and miR‐34a GA+AA [33] demonstrated significant associations with coagulation factor abnormalities. Given the established role of thrombophilia in RIF and miscarriage [57, 58, 59, 60, 61, 62], these findings could support the use of low‐dose aspirin or anticoagulants in genetically susceptible patients, although further clinical trials are needed.

Moreover, the immune milieu is another potential therapeutic target. The influence of E2 on immune tolerance via IL‐35 [63] and the interaction of miRNA polymorphisms with cytokine production (e.g., transforming growth factor beta—TGF‐β, IL‐10, IL‐35) highlight the importance of immune modulation. Variants impacting miR‐449 [32] and miR‐222 [31] may alter inflammatory signaling, supporting future exploration of immunomodulatory therapies such as corticosteroids or intravenous immunoglobulin (IVIg) in patients with RIF and relevant polymorphisms.

Thus, while current studies focus primarily on diagnostic and prognostic associations, expanding the translational scope of miRNA polymorphism research to include personalized therapeutic interventions represents a promising future direction. These genetic insights may one day enable the development of individualized treatment protocols based on genotype‐specific profiles, ultimately improving implantation outcomes and pregnancy rates in patients with RIF.

To accurately interpret the role of miRNAs in RIF, it is crucial to distinguish between two key aspects of miRNA biology: (1) miRNA polymorphisms, typically SNPs, which are inherited genetic variations that remain stable throughout an individual's lifetime and (2) miRNA expression levels, which are dynamic and responsive to environmental, hormonal, and epigenetic factors. Polymorphisms can affect miRNA biogenesis, maturation, or target binding, serving as static genetic risk markers for RIF [64]. Conversely, miRNA expression is modulated by various environmental exposures such as inflammation, smoking, nutritional status, and endocrine‐disrupting chemicals (EDCs) [65]. These expression changes are potentially reversible and thus represent promising therapeutic targets [66].

While miRNA polymorphisms represent stable genetic predispositions, growing evidence highlights that miRNA expression is also highly dynamic and responsive to environmental and epigenetic factors. Inflammatory states, oxidative stress (OS), and exposure to pollutants or EDCs can significantly alter miRNA profiles. For instance, exposure to diesel exhaust particles has been shown to disrupt miRNA expression in human airway cells, affecting gene networks involved in inflammation and disease [67]. Similarly, EDCs such as bisphenol A (BPA) and phthalates have been associated with dysregulation of miRNAs in reproductive tissues. BPA exposure was linked to increased miR‐146a levels in placental cells, correlating with adverse pregnancy outcomes [68], while phthalates have been shown to modulate expression of the miR‐34 family, which governs critical implantation‐related processes such as apoptosis and cell cycle regulation [69].

Lifestyle factors also play a pivotal role in modulating miRNA expression and may interact with genetic predispositions to influence reproductive outcomes. Smoking, for example, has been linked to the upregulation of pro‐inflammatory miRNAs such as miR‐21 and miR‐146a, which may impair ER and increase the risk of IF [70]. In contrast, diets rich in fruits, vegetables, and omega‐3 fatty acids are associated with increased expression of protective miRNAs such as miR‐let‐7a and miR‐328 and a reduction in pro‐inflammatory miRNAs [70].

These findings underscore the relevance of modifiable environmental and nutritional exposures in shaping the miRNA landscape. Integrating such factors with genetic screening may enhance clinical understanding of RIF pathophysiology and support the development of personalized interventions aimed at improving implantation success.

### 4.1 Strengths and Limitations of the Study

To the authors' best knowledge, this is the first systematic review conducted on the role of miRNA polymorphisms in RIF, with the included case–control studies demonstrating intermediate‐to‐high methodological quality. A noteworthy strength is the uniformity of study settings, as all participants were enrolled from a single fertility center, specifically CHA Bundang Medical Center in Seongnam, South Korea, ensuring consistent clinical protocols and laboratory techniques.

However, several limitations must be considered when interpreting the findings. First, the exact molecular mechanisms by which the identified miRNA polymorphisms contribute to RIF remain to be fully elucidated. Second, the entire study population was comprised exclusively of Korean women, which limits the generalizability of these findings to broader populations. Ethnic differences in both miRNA polymorphism frequencies and expression levels are well‐documented in the literature. Third, none of the reviewed studies adjusted for potential confounding variables such as patient comorbidities, IVF protocols, or environmental exposures, which may interact with genetic predispositions to affect implantation success. Fourth, the sample sizes in the included studies were relatively modest, potentially limiting statistical power and the ability to detect less prevalent polymorphisms. Future investigations should consider larger, multi‐ethnic cohorts and incorporate environmental and clinical data to allow for more comprehensive and generalizable conclusions.

Rawlings‐Goss et al. [71] identified 31 miRNA variants with significant allele frequency differences between African and non‐African populations, including a novel deletion in hsa‐mir‐4640 with functional implications for mRNA targeting. This emphasizes the influence of population‐specific architectures on disease susceptibility. Additionally, differential expression patterns of 351 miRNAs across individuals of African and European ancestry have been observed, potentially contributing to population‐specific disease phenotypes and treatment responses [72]. Gong et al. [73] investigated miRNA expression in breast cancer tissues from women of African and European ancestry. The researchers identified several miRNAs that were differentially expressed between the two groups, with some miRNAs showing distinct expression patterns in E2 receptor‐negative (ER−) tumors. For instance, miR‐105‐5p and miR‐767‐5p were downregulated in African American women compared to European American women, while miR‐187‐3p and miR‐937‐3p were upregulated in African American women.

Xu et al. [74] conducted a meta‐analysis focusing on Asian populations and found that certain miRNA polymorphisms, such as rs2910164 in miR‐146a, rs11614913 in miR‐196a2, and rs3746444 in miR‐499, exhibited varying associations with cancer risk across different Asian subpopulations, highlighting the influence of genetic background on disease susceptibility. Similarly, Alimena et al. [75] investigated serum miRNA profiles across diverse racial and ethnic groups and discovered significant variations in miRNA expression levels, emphasizing the necessity for population‐specific validation of miRNA‐based biomarkers. Sonehara et al. [76] conducted a comprehensive analysis of miRNA expression quantitative trait loci (miRNA‐eQTLs) in a Japanese cohort, identifying 1275 cis‐miRNA‐eQTL variants for 40 miRNAs. Notably, 25 of these miRNAs with eQTLs were unreported in European studies, and five had lead variants monomorphic in European populations, highlighting the presence of population‐specific genetic architectures influencing miRNA expression. Similarly, a meta‐analysis by Yan et al. [77] revealed that the association between miRNA‐196a2 rs11614913 polymorphism and cervical cancer risk among different ethnic groups, highlighting the necessity for population‐specific studies.

Therefore, while the current findings provide valuable insights into miRNA polymorphisms associated with RIF in Korean women, caution should be exercised when extrapolating these results to other populations. Future research should aim to validate these associations in diverse ethnic cohorts to enhance the translational relevance and applicability of miRNA‐based diagnostics and therapeutic interventions in reproductive medicine.

## 5 Conclusions

In conclusion, this systematic review highlighted that certain frequencies and combinations of genotypes and alleles of miRNAs are linked with either a protective role or an increased risk of RIF. Interactions with specific parameters can negatively impact pregnancy progression and may prompt further studies to explore miRNAs as potential prognostic or diagnostic biomarkers for RIF. This manuscript clarifies the essential evidence and supports the development of future randomized controlled trials (RCTs) to effectively translate findings into clinical practice by designing management strategies for women diagnosed with RIF. Prospectively, the integration of advanced transcriptomic technologies promises to refine our understanding of ER and its disruption in RIF. Techniques such as single‐cell RNA sequencing (scRNA‐seq) and spatial transcriptomics offer unprecedented resolution in mapping cell‐type‐specific gene expression and spatial organization within the endometrial microenvironment. Additionally, the profiling of circulating miRNAs from blood or uterine fluid may serve as a minimally invasive biomarker approach for patient stratification and monitoring treatment response. As these tools become increasingly accessible, they are expected to complement genomic association studies by unveiling dynamic regulatory networks, improving diagnostic precision, and informing personalized therapeutic strategies in RIF.

## Ethics Statement

The authors have nothing to report.

## Consent

The authors have nothing to report.

## Conflicts of Interest

The authors declare no conflicts of interest.

## Supporting information


Data S1.

Data S2.

Data S3.

Data S4.

## Data Availability

Data sharing is not applicable to this article, as no datasets were generated or analyzed during the current study.
