# Supplementary material for: A Systematic Evidence‐Based Review Regarding miRNA Polymorphisms in Recurrent Implantation Failure
Source: Reprod Med Biol. 2025 Jul 30;24(1):e12670. doi: 10.1002/rmb2.12670 (PMC12309981; doi:10.1002/rmb2.12670)
Supplement: Supplementary file 1 — Data S1. [file RMB2-24-e12670-s003.docx]

**Supplementary File 1.** Detailed searching strategies

**Primary keywords**

MicroRNA, miRNA, pre-miRNA, pri-miRNA, miR, polymorphisms, RIF

**1. PubMed-MEDLINE – United States (NLM, 1996)**

***1.1. Extensive searching string:***

((((((MicroRNA) OR (miRNA)) OR (pre-miRNA)) OR (pri-miRNA)) OR (miR)) AND (polymorphisms)) AND (RIF) Filters: Humans, English, Female, MEDLINE, from 2014/1/1 - 2024/9/1

(("microrna s"[All Fields] OR "micrornas"[MeSH Terms] OR "micrornas"[All Fields] OR "microrna"[All Fields] OR ("micrornas"[MeSH Terms] OR "micrornas"[All Fields] OR "mirna"[All Fields] OR "mirnas"[All Fields] OR "mirna s"[All Fields]) OR ("micrornas"[MeSH Terms] OR "micrornas"[All Fields] OR ("pre"[All Fields] AND "mirna"[All Fields]) OR "pre mirna"[All Fields]) OR ("micrornas"[MeSH Terms] OR "micrornas"[All Fields] OR ("pri"[All Fields] AND "mirna"[All Fields]) OR "pri mirna"[All Fields]) OR ("med int rev"[Journal] OR "manag int rev"[Journal] OR "mir"[All Fields])) AND ("polymorphic"[All Fields] OR "polymorphics"[All Fields] OR "polymorphism s"[All Fields] OR "polymorphism, genetic"[MeSH Terms] OR ("polymorphism"[All Fields] AND "genetic"[All Fields]) OR "genetic polymorphism"[All Fields] OR "polymorphism"[All Fields] OR "polymorphisms"[All Fields]) AND ("rev infect"[Journal] OR "rif"[All Fields])) AND ((medline[Filter]) AND (humans[Filter]) AND (female[Filter]) AND (2014/1/1:2024/9/1[pdat]) AND (english[Filter]))

***1.2. Filters applied for fields:***

PUBLICATION DATE – 2014/1/1 to 2024/9/1

SPECIES – Humans

ARTICLE LANGUAGE – English

SEX – Female

OTHER – MEDLINE

**2. Web of Science^TM^ (WOS) (Clarivate Analytics, 1997)**

***2.1. Extensive searching string:***

((((((ALL=(MicroRNA)) OR ALL=(miRNA)) OR ALL=(pre-miRNA)) OR ALL=(pri-miRNA)) OR ALL=(miR)) AND ALL=(polymorphisms)) AND ALL=(RIF)

***2.2. Filters applied for fields:***

PUBLICATION YEARS – 2014-2024

DOCUMENT TYPES – Article

LANGUAGE – English

WEB OF SCIENCE INDEX – Science Citation Index Expanded (SCI-EXPANDED)

**3. Scopus (Elsevier, 2004)**

***3.1. Extensive searching string:***

TITLE-ABS-KEY ( microrna OR mirna OR pre-mirna OR pri-mirna OR mir ) AND TITLE-ABS-KEY ( polymorphisms ) AND TITLE-ABS-KEY ( rif ) AND ( LIMIT-TO ( PUBYEAR , 2016 ) OR LIMIT-TO ( PUBYEAR , 2019 ) OR LIMIT-TO ( PUBYEAR , 2020 ) OR LIMIT-TO ( PUBYEAR , 2023 ) OR LIMIT-TO ( PUBYEAR , 2024 ) ) AND ( LIMIT-TO ( DOCTYPE , "ar" ) ) AND ( LIMIT-TO ( PUBSTAGE , "final" ) ) AND ( LIMIT-TO ( SRCTYPE , "j" ) ) AND ( LIMIT-TO ( LANGUAGE , "English" ) )

***3.2. Filters applied for fields:***

YEAR – 2014-2024

DOCUMENT TYPE – Article

PUBLICATION STAGE – Final

SOURCE TYPE – Journal

LANGUAGE – English

**4. Excerpta Medica dataBASE (EMBASE) (Elsevier, 1947)**

***4.1. Extensive searching string:***

(**'microrna'**/exp OR **microrna** OR **'mirna'**/exp OR **mirna** OR **'pre mirna'** OR **'pri mirna'** OR **mir**) AND **polymorphisms** AND **rif** AND [article]/lim AND [english]/lim AND [female]/lim AND [humans]/lim AND [embase]/lim AND [01-01-2014]/sd NOT [02-06-2024]/sd

***4.2. Filters applied for fields:***

MAPPING – Map to preferred term in Emtree, Search also as free text in all fields, Explode using narrower Emtree terms, Search as broadly as possible

DATE – 01-01-2014 to 01-09-2024

SOURCES – Embase

QUICK LIMITS – Humans

PUBLICATION TYPES – Article

LANGUAGES – English

GENDER – Female
