# Supplementary material for: A Systematic Evidence‐Based Review Regarding miRNA Polymorphisms in Recurrent Implantation Failure
Source: Reprod Med Biol. 2025 Jul 30;24(1):e12670. doi: 10.1002/rmb2.12670 (PMC12309981; doi:10.1002/rmb2.12670)
Supplement: Supplementary file 2 — Data S2. [file RMB2-24-e12670-s001.docx]

**Supplementary File 2.** Overall tabular summarization of studies per year of publication and database

|  | **Databases** | | | |
| --- | --- | --- | --- | --- |
| **Year of publication** | **PubMed-MEDLINE** | **WOS** | **Scopus** | **EMBASE** |
| **2014** | **-** | **-** | **-** | **-** |
| **2015** | **-** | **1** | **-** | **-** |
| **2016** | **1** | **1** | **1** | **1** |
| **2017** | **-** | **-** | **-** | **-** |
| **2018** | **-** | **1** | **-** | **-** |
| **2019** | **3** | **3** | **2** | **2** |
| **2020** | **1** | **2** | **-** | **1** |
| **2021** | **-** | **-** | **-** | **-** |
| **2022** | **-** | **-** | **-** | **-** |
| **2023** | **1** | **1** | **1** | **1** |
| **2024** | **1** | **2** | **-** | **-** |
| **Cumulative** | **7** | **11** | **4** | **5** |
| **Total** | **27** | | | |
