# Supplementary material for: A Systematic Evidence‐Based Review Regarding miRNA Polymorphisms in Recurrent Implantation Failure
Source: Reprod Med Biol. 2025 Jul 30;24(1):e12670. doi: 10.1002/rmb2.12670 (PMC12309981; doi:10.1002/rmb2.12670)
Supplement: Supplementary file 3 — Data S3. [file RMB2-24-e12670-s002.docx]

**Supplementary File 3.** PICO model

| **Parameter** | **Inclusion criteria** | **Exclusion criteria** | **Data extraction** |
| --- | --- | --- | --- |
| **Patient, Population or Problem** | -Adult females (18-40 yo)  -Normal menstrual cycle length (21-35 days)  -Approximately 3 months of no hormonal or intrauterine contraception use  -Tubal or male-related factor infertility  -Normal endocrine profile parameters (FSH and LH < 10 mIU/mL, E2 < 50 pg/mL) on day 3  -BMI ranging from 18.5-24.9 kg/m^2^ | RIF due to one or multiple causes:  -Anatomical  -Chromosomal  -Hormonal  -Infections  -Autoimmune  -Thrombotic | -Author’s first name and year of publication  -Country  -Study settings  -Study design  -Population and sample size |
| **Intervention or Exposure** | Molecular biology protocols for identifying miRs polymorphisms in RIF patients | Identification of miRs in RIF group with or without a Control/Healthy individuals group | - |
| **Comparison or Control** | Adult females (18-40 yo) with RIF undergoing miR identification through molecular biology techniques | A single group of analysis without RIF and/or Control/Healthy individuals group | - |
| **Outcome** | PCR-based analyses | Reproductive outcomes | -Genotype frequencies  -Genotype combinations  -Allele combinations  -Coagulation factors  -Hormonal factors  -Renal factors |

yo – years old, FSH – follicle-stimulating hormone, LH – luteinizing hormone, E2 – estradiol, BMI – body mass index, RIF – recurrent implantation failure, PCR – polymerase chain reaction
