# Supplementary material for: A Systematic Evidence‐Based Review Regarding miRNA Polymorphisms in Recurrent Implantation Failure
Source: Reprod Med Biol. 2025 Jul 30;24(1):e12670. doi: 10.1002/rmb2.12670 (PMC12309981; doi:10.1002/rmb2.12670)
Supplement: Supplementary file 4 — Data S4. [file RMB2-24-e12670-s004.docx]

**Supplementary File 4.** Assessment phases of studies

**First list of manuscripts non- and eligible arranged in chronological order**

| **Author’s first name and year** | **Article title** | **Eligible or not eligible?** | **Reason** |
| --- | --- | --- | --- |
| Nicolaidou et al., 2015 | Gene expression changes in HLA mismatched mixed lymphocyte cultures reveal genes associated with allorecognition | **Not eligible** | **Experimental** |
| Cho et al., 2016 | Association of miR-146aC>G, miR-149C>T, miR-196a2T>C, and miR-499A>G polymorphisms with risk of recurrent implantation failure in Korean women | **Eligible** | **Included in the manuscript** |
| Gonçalves et al., 2018 | Gene Co-expression Analysis Indicates Potential Pathways and Regulators of Beef Tenderness in Nellore Cattle | **Not eligible** | **Animal model** |
| Lee et al., 2019 | Associations between microRNA (miR-25, miR-32, miR-125, and miR-222) polymorphisms and recurrent implantation failure in Korean women | **Eligible** | **Included in the manuscript** |
| Lee et al., 2019 | Association Between miR-605A>G, miR-608G>C, miR-631I>D, miR-938C>T, and miR-1302-3C>T Polymorphisms and Risk of Recurrent Implantation Failure | **Eligible** | **Included in the manuscript** |
| Ryu et al., 2019 | The association of AGO1 (rs595961G>A, rs636832A>G) and AGO2 (rs11996715C>A, rs2292779C>G, rs4961280C>A) polymorphisms and risk of recurrent implantation failure | **Not eligible** | **Lack of miR association with RIF** |
| Kim et al., 2020 | Association of miR-27aA>G, miR-423C>a, miR-449bA>G, and miR-604A>G Polymorphisms with Risk of Recurrent Implantation Failure | **Eligible** | **Included in the manuscript** |
| Lee et al., 2020 | Association between microRNA machinery gene polymorphisms and recurrent implantation failure | **Not eligible** | **Lack of miR association with RIF** |
| Lee et al., 2023 | Genetic Correlation of miRNA Polymorphisms and STAT3 Signaling Pathway with Recurrent Implantation Failure in the Korean Population | **Eligible** | **Included in the manuscript** |
| Fathi et al., 2024 | Impact of miRNAs in the pathoetiology of recurrent implantation failure | **Not eligible** | **Review** |
| Fan et al., 2024 | Unraveling the H19/GAS1 axis in recurrent implantation failure: A potential biomarker for diagnosis and insight into immune microenvironment alteration | **Not eligible** | **Network analysis** |

**Second list of manuscripts eligible arranged in chronological order**

| **Author’s first name and year** | **Article title** | **Eligible or not eligible?** | **Reason** |
| --- | --- | --- | --- |
| Cho et al., 2016 | Association of miR-146aC>G, miR-149C>T, miR-196a2T>C, and miR-499A>G polymorphisms with risk of recurrent implantation failure in Korean women | **Eligible** | **Included in the manuscript** |
| Lee et al., 2019 | Associations between microRNA (miR-25, miR-32, miR-125, and miR-222) polymorphisms and recurrent implantation failure in Korean women | **Eligible** | **Included in the manuscript** |
| Lee et al., 2019 | Association Between miR-605A>G, miR-608G>C, miR-631I>D, miR-938C>T, and miR-1302-3C>T Polymorphisms and Risk of Recurrent Implantation Failure | **Eligible** | **Included in the manuscript** |
| Kim et al., 2020 | Association of miR-27aA>G, miR-423C>a, miR-449bA>G, and miR-604A>G Polymorphisms with Risk of Recurrent Implantation Failure | **Eligible** | **Included in the manuscript** |
| Lee et al., 2023 | Genetic Correlation of miRNA Polymorphisms and STAT3 Signaling Pathway with Recurrent Implantation Failure in the Korean Population | **Eligible** | **Included in the manuscript** |

| **Third list of manuscripts excluded, and the reason, arranged in chronological orderAuthor’s first name and year** | **Article title** | **Reason for Exclusion** |
| --- | --- | --- |
| Nicolaidou et al., 2015 | Gene expression changes in HLA mismatched mixed lymphocyte cultures reveal genes associated with allorecognition | Excluded due to experimental in vitro model focusing on allorecognition mechanisms without direct analysis of miRNA polymorphisms or RIF. |
| Gonçalves et al., 2018 | Gene Co-expression Analysis Indicates Potential Pathways and Regulators of Beef Tenderness in Nellore Cattle | Excluded because the study was conducted on an animal model (cattle), not involving human subjects or relevant polymorphisms data related to RIF. |
| Ryu et al., 2019 | The association of AGO1 (rs595961G>A, rs636832A>G) and AGO2 (rs11996715C>A, rs2292779C>G, rs4961280C>A) polymorphisms and risk of recurrent implantation failure | Excluded due to lack of demonstrated functional association between the investigated polymorphisms and miRNA pathways relevant to RIF. |
| Lee et al., 2020 | Association between microRNA machinery gene polymorphisms and recurrent implantation failure | Excluded because although polymorphisms were studied, the analysis did not establish a significant relationship with miRNA function or RIF pathogenesis. |
| Fathi et al., 2024 | Impact of miRNAs in the pathoetiology of recurrent implantation failure | Excluded because it is a narrative review article without original experimental or genetic data relevant to polymorphisms in miRNA machinery genes. |
| Fan et al., 2024 | Unraveling the H19/GAS1 axis in recurrent implantation failure: A potential biomarker for diagnosis and insight into immune microenvironment alteration | Excluded because the study employed network analysis and biomarker identification without direct investigation of SNP polymorphisms or functional miRNA genetics in RIF. |
